# Effects of sulforaphane intake on processing speed and negative moods in healthy older adults: Evidence from a randomized controlled trial

**DOI:** 10.3389/fnagi.2022.929628

**Published:** 2022-07-29

**Authors:** Rui Nouchi, Qingqiang Hu, Yusuke Ushida, Hiroyuki Suganuma, Ryuta Kawashima

**Affiliations:** ^1^Department of Cognitive Health Science, Institute of Development, Aging and Cancer, Tohoku University, Sendai, Japan; ^2^Smart Aging Research Center, Tohoku University, Sendai, Japan; ^3^Innovation Division, KAGOME CO., LTD., Nasushiobara, Japan; ^4^Department of Functional Brain Imaging, Institute of Development, Aging and Cancer, Tohoku University, Sendai, Japan

**Keywords:** sulforaphane, nutrition, processing speed, mood state, biomarker

## Abstract

**Background:**

Recent studies have reported that sulforaphane (SFN) intake with cognitive training had positive effects on cognitive functions. However, it is still unknown whether SFN intake alone has beneficial effects on cognition as well as mood. We investigated whether a SFN intake intervention improved cognitive performance and mood states in healthy older adults.

**Methods:**

In a 12-week, double-blinded, randomized controlled trial (RCT), we randomly assigned 144 older adults to a SFN group or a placebo group. We asked the participants to take a supplement (SFN or placebo) for 12 weeks. We measured several cognitive functions, mood states, and biomarkers before and after the intervention period.

**Results:**

The SFN group showed improvement in processing speed and a decrease in negative mood compared to the placebo group. In addition, the SFN group exhibited a higher SFN-*N*-acetyl-L-cysteine (NAC) level compared to the placebo group. However, there were no significant results in other biomarkers of oxidant stress, inflammation, or neural plasticity.

**Discussion:**

These results indicate that nutrition interventions using SFN can have positive effects on cognitive functioning and mood in healthy older adults.

## Introduction

Aging is a global phenomenon and negatively affects cognitive functioning (Salthouse, 2019) and mood state (Karel, 1997). Cognitive decline and negative mood are associated with challenges in everyday behavior and social communication (de Paula et al., 2015). For example, older adults with lower cognitive functioning and depressive symptoms engaged in fewer activities of daily living (ADLs) (de Paula et al., 2015). Thus, it is vital to enhance cognitive functioning and mood state in this population.

Nutrients are critical for better cognitive and mental health in older adults. Phytochemicals contain color, aroma, and flavor and are found in fruits, vegetables, grains, beans, and other plants; examples include carotenoids, flavonoids, and isothiocyanates. Their consumption generally provides beneficial health effects (Tan and Norhaizan, 2019; Rapposelli et al., 2022). Several randomized controlled trials (RCTs) found that carotenoids and flavonoids had benefits on cognitive functions and mood states (Nouchi et al., 2020b; Tanprasertsuk et al., 2021; Yagi et al., 2021; Park et al., 2022). However, few studies have investigated the beneficial outcomes of isothiocyanate on cognitive functioning and mood state in healthy older adults (Nouchi et al., 2021a). Hence, we aimed to examine the influence of isothiocyanate on cognitions and mood state in healthy older adults.

Sulforaphane (SFN) is an isothiocyanate found in cruciferous vegetables such as cauliflower and broccoli. Only two RCTs with human participants have explored the effects of SFN intake on cognitive functioning and mood (Shiina et al., 2015; Nouchi et al., 2021a). SFN intake has shown beneficial effects in cognitive performance and reduced symptoms in schizophrenia patients (Shiina et al., 2015). Further, another study involving healthy older adults revealed that cognitive training combined with SFN enhanced several cognitive functions (Nouchi et al., 2021a). In addition, previous animal studies have found that SFN reduced depressive behaviors in mice (Wu et al., 2016; Zhang et al., 2017; Ferreira-Chamorro et al., 2018). Hence, SFN intake could have beneficial effects on cognitive functioning and mood state. However, it is still unknown whether SFN alone could improve cognitive functioning and mood state in healthy older adults. As such, we conducted an RCT to examine the benefits of SFN intake on cognitive functioning and mood state in healthy older adults.

For several reasons, we hypothesized that SFN intake would improve processing speed, working memory, and negative mood state. First, previous studies using older adults have demonstrated that SFN intake, combined with cognitive training, boosted processing speed performance, and working memory capacity (Nouchi et al., 2021a). Second, the intake of fruits and vegetables has been correlated with lower negative mood in older adults (Ford et al., 2013). Taken together, we expected that SFN intake would heighten the performance of processing speed and the capacity of working memory and reduce negative mood.

To consider the biological mechanism of the effects of SFN, we measured blood biomarkers [oxidative stress: heme oxygenase-1 (HO-1) and glutathione S-transferase (GST)], inflammation [tumor necrosis factor-α (TNF-α)], neural plasticity [brain derived neurotrophic factor (BDNF)], and a urine biomarker [SFN absorption: sulforaphane *N*-acetyl-L-cysteine (SFN-NAC)]. Previous animal studies have reported the effects of SFN on the biomarkers of oxidative stress, inflammation, and neural plasticity (Dwivedi et al., 2016; Wang et al., 2016; Jhang et al., 2018). In addition, prior studies with human participants found that SFN intake led to changes in the SFN-NAC level (Nouchi et al., 2021a). Thus, we expected that SFN intake would alter the levels of HO-1, GST, TNF-α, and BDNF. Moreover, we expected the SFN intake group to exhibit a greater SFN absorption level compared to the placebo group, whose members took placebo supplements without SFN (please see “Sulforaphane and placebo supplements” section).

## Materials and methods

### Design and setting of the randomized controlled trial

We carried out an RCT from March 2021 to June 2021 in Tokyo, Japan. The protocol was approved by the Ethics Committee of KAGOME CO., LTD. and the Ethics Committee of Nihonbashi Cardiology Clinic. Further, we registered the RCT with the University Hospital Medical Information Network Clinical Trial Registry (UMIN000042666).

**FIGURE 1:**
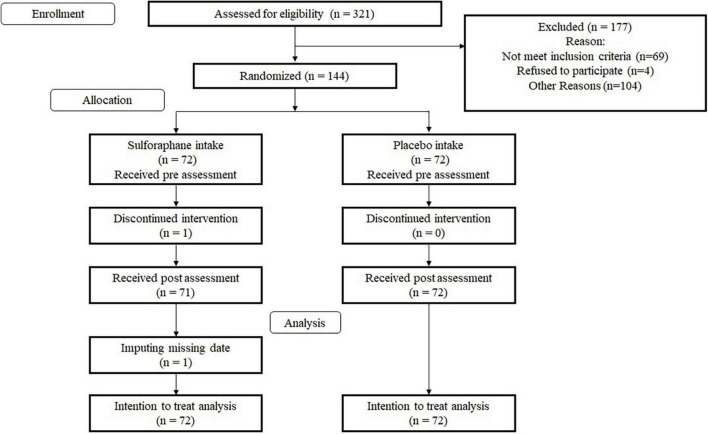
CONSORT diagram.

This RCT was a double-blinded RCT using an SFN group and a placebo group. We blinded participants and testers to the specific research hypotheses related to cognitive functions and mood and their group membership. We used several cognitive, emotional, and biological measures. The primary outcome measures were the performance of a processing speed test and working memory scores. Further, we employed the Consolidated Standards of Reporting Trials statement (see Supplementary Table 1)^1^ to report the study structure. The RCT design is shown in Figure 1.

### Participants

We recruited participants *via* e-mail from a pool of individuals listed by L. Smile Co., Ltd. Subsequently, 321 individuals were invited to participate in a screening. After receiving written informed consent from each participant, the testers checked whether they were eligible for the study. All participants underwent the screening, which included a blood test, the Japanese version Mini-Mental State Examination (MMSE-J) (Folstein et al., 1975), the Frontal Assessment Battery at bedside (FAB) (Dubois et al., 2000), the Japanese Reading Ability Test (JART) (Matsuoka et al., 2006), and the Geriatric Depression Scale-15 (GDS) (Sugishita et al., 2017). We excluded 69 participants based on the results of the screening. Four individuals refused to participate, and 104 did not take part due to other reasons. Finally, 144 individuals (73 men, 71 women) participated. Average age of all participants was 66.82 years (standard deviation, *SD* = 4.29). One person in the SFN group did not complete the intervention due to an inability to adhere to the study’s schedule. Table 1 shows the baseline characteristics in each group.

**TABLE 1:** The baseline characteristics in each group.

	SFN	PL
Age	66.89	66.75
	(4.44)	(4.17)
MMSE-J	28.67	28.71
	(1.20)	(1.13)
FAB	15.10	15.13
	(1.94)	(1.88)
JART	109.78	109.58
	(9.06)	(8.04)
GDS	1.42	1.43
	(1.30)	(1.34)

SFN, sulforaphane; PL, placebo; MMSE, Japanese version’s Mini-Mental State Examination; FAB, Frontal Assessment Battery at bedside; JART, Japanese Reading Test; GDS, Geriatric Depression Scale.

### Inclusion and exclusion criteria

Based on our earlier studies (Nouchi et al., 2012, 2016a,2016b; Nozawa et al., 2015; Kawata et al., 2022), we set the following inclusion criteria: participants should be healthy native Japanese (60–80 years of age). They agreed to receive medical and physical checkups and to have samples of their blood taken. The exclusion criteria were as follows: Participants (1) were suffering from mental illness, diabetes, neurological disease, heart (cardiac) disease, or another serious illness; (2) using medications that interfere with cognitive functioning (including benzodiazepines, antidepressants, or other central nervous system agents); (3) had a history of serious illness and problems that precluded them from participation; (4) had an MMSE-J score of less than 26; (5) had a GDS score higher than 5; (6) had an FAB score of less than 12; (7) had an IQ score lower than 85; (8) had food allergy concerns (especially allergies to broccoli, milk, eggs, wheat, buckwheat, peanuts, shrimp, and crab); (9) drank more than 60 g of alcohol per day on average for a week; (10) had taken part in other intervention-based studies within the past 2 months; (11) were unable to refrain from using medicines and health foods (including supplements, foods for specified health use, foods with health claims, aojiru, energy drinks) during the intervention period; or (12) were unable to ingest the SFN or placebo supplements as instructed during the intervention period and to fill out the life diary. It noted that aojiru is a powdered health food in Japan, which made mainly from kale, a cruciferous vegetable contained glucoraphanin. Finally, we excluded participants whom the principal investigator judged to be inappropriate for the study.

### Sample size calculation

Using symbol search (SS), a prior study using older adults found that 12 weeks of SFN intake improved processing speed performance (Nouchi et al., 2021a). In the previous study, the effect size was a medium (Cohen’ d = 0.48, the SFN group vs. the control group). Therefore, we assumed that the SFN group would have a greater effect in SS compared to the placebo group in this study. The sample size was calculated using G*power which was a tool to estimate a sample size (Faul et al., 2009). We used the general liner model [processing speed score at the baseline, MMSE-J, gender, and age as the covariates, a one-tailed test, α = 0.025, power = 0.80, effect size (*f*^2^) = 0.05]. We computed the *f*^2^ based on the abovementioned study (Nouchi et al., 2021a). Finally, we set 144 as the sample size.

### Randomization

After recruitment, we randomly assigned the 144 eligible participants into the two groups using JMP software, Version 5.01 (SAS Institute, Cary, NC). The cognitive functions were the primary outcome measures, with sex affecting several aspects of cognitive performance (McCarrey et al., 2016). Thus, we stratified the participants based on sex; in addition, we adjusted other attributes (age, pre-MMSE-J) as moderating factors, with an allocation ratio of 1:1.

### Sulforaphane and placebo supplements

The participants took three capsules of the SFN or placebo supplements per day for 12 weeks. Participants took the three capsules with water at once. The SFN supplements contained 30 mg of glucoraphanin, which is converted to SFN *via* gut microbial thioglucosidase (Fahey et al., 2012). The placebo supplements contained 0 mg of glucoraphanin (Fahey et al., 2012). KAGOME CO., LTD. provided both the supplements (Nagoya, Japan). Researchers, participants, and testers were blinded to the supplements until the study was completed.

### Cognitive functioning

Firstly, we assessed the participants’ cognitive status using the MMSE-J, FAB, and JART during the screening. To obtain a comprehensive picture of cognitive status, we used the MMSE-J which gauges individuals’ memory, attention, language, and visuospatial abilities (Folstein et al., 1975). Moreover, to ascertain participants’ reading ability and IQ, we used JART (Matsuoka et al., 2006), the Japanese version of the National Adult Reading Test. JART depicts 25 kanji (Chinese characters) compound words, and the participants were asked to write down the pronunciation of each kanji compound word. The FAB is a simple screening test that measures frontal lobe functioning (Dubois et al., 2000).

We examined executive functioning, processing speed, visual, and verbal episodic memory performance, verbal short-term memory performance, and a capacity of working memory using the standardized cognitive tests. In this study, processing speed was measured by SS and digit symbol coding (Cd) from the Wechsler Adult Intelligence Scale, 3rd edition (WAIS-III) (Wechsler, 1997). In the SS, participants were asked to check whether the target symbols existed in the other symbol pool. In the Cd, participants were asked to draw the symbol corresponding numbers. For executive functioning, we used Trail making test (TMT) (Japan Society for Higher Brain Dysfunction, Brain function test committee, 2019) and two types of Stroop tests such as Stroop task (ST) and reverse Stroop task (rST) (Hakoda and Watanabe, 2004). In the TMT, participants were asked to link numbers or letters. Short-term memory was measured by digit span (DS) (Wechsler, 1997). The digit span requires participants to memorize the series of numbers in the same order or in the reverse order. Working memory was measured by letter number sequence (LNS) (Wechsler, 1997). In LNS, participants listened to the combination of numbers and letters. Then, they were asked to recall numbers and letters in the ascending order. For episodic memory, we used logical memory (LM), design memory (DM), visual paired associates (VPA), and visual reproduction (VR) from the Wechsler Memory Scale-Revised (WMS-R) (Wechsler, 1987). The LM was the verbal episodic memory task. In the LM, participants were asked to memorize the short story and recall the story immediately and after about 30 min delay. The DM, the VPA, and the VR were visual episodic memory task. Participants were required to memorize the abstract figures in the DM, the pairs of figures and colors in the VPA, and geometric shapes in the VR. The details of each cognitive measure were shown in Supplementary File 1.

### Emotional state

For the participant’s emotional state, we used a short version of the Profile of Mood State Second Edition (POMS2) (Heuchert and McNair, 2012; Yokoyama and Watanabe, 2015). In the POMS2, participants were asked to rate the six emotional sates [depression–dejection (D), confusion–bewilderment (C–B), friendliness (F), vigor–activity (V), fatigue–inertia (F–I), anger–hostility (A–H), tension–anxiety (T–A)] in the prior week. The total mood disturbance (TMD) scores was estimated using the six emotional states (Nouchi et al., 2021a).

We measured quality of life using the World Health Organization-Five WellBeing Index (WHO-5). In WHO-5, participants were asked to rate their mental wellbeing within 2 weeks. A higher score in WHO-5 means good mental wellbeing (Nouchi et al., 2021a).

### Blood biomarkers

We measured antioxidant response (GST and HO-1), neuroplasticity (BDNF), and the neuroinflammation (TNF-α) blood parameter. Experienced nurses collected blood through venipuncture in 5 mL separator tubes for serum and 5 mL EDTA tubes for plasma (Nipro Corporation, Osaka, Japan). The obtained serum and plasma samples were aliquoted and stored at –80°C.

BDNF has three different forms such as prodomain BDNF, mature BDNF, and precursor BDNF (Hempstead, 2015). The mature BDNF has neurotrophic and neuroprotective functions. In this study, we measured the serum levels of mature BDNF using a mature BDNF rapid ELISA kit (Biosensis, Thebarton, Australia). The serum levels of TNF-α were measured with and a Quantikine HS ELISA kit (R&D systems, Minneapolis, MN, United States), respectively, following the manufacturer’s instructions. The enzyme activity of GST was determined with 1-chloro-2,4-dinitrobenzene as the substrate, as described previously (Kikuchi et al., 2015). The plasma level of HO-1 was determined using a StressXpress HO-1 ELISA kit (StressMarq Biosciences, Victoria, BC, Canada) following the manufacturer’s instructions.

### Urine biomarker

We used the excreted level of SFN-NAC from urine samples. The SFN-NAC is a main metabolite of SFN. The urine sample was collected each participant 10–12 h after the intake of the SFN or placebo at the last day. The time was when urinary excretion of isothiocyanate metabolites reached a maximum level (Ushida et al., 2015). The urinary levels of SFN-NAC were determined using a LC-MS/MS system. In this study, the urinary levels of SFN-NAC were standardized to creatinine levels (Nouchi et al., 2021a). The details of the urine analysis were described in Supplementary File 2.

### Analyses

We performed all analyses using the software R (Version 4.12). One person in the SFN group did not take the tests after the intervention period. Therefore, all cognitive, emotional, and biomarker measures of the one participant were missing data. First, we imputed missing data using the multiple imputation method (predictive mean matching, *m* = 20) *via* the “mice” function in the mice package (van Buuren and Groothuis-Oudshoorn, 2011). Next, we calculated the changes in scores for cognitive functioning, emotional states, and biomarkers (the post-intervention score minus the pre-intervention score). Second, we used a permutation general linear model (GLM) for all changes in scores in cognition, emotional states, and biomarkers. In the model, the change score in cognition, emotion or biomarkers was the dependent variable, and the supplement factor (SFN or placebo) was the independent variable. In the GLM for measuring cognitive functioning, we used the pre-scores for the dependent variable, MMSE score, age, and sex as covariates. In the GLM for measuring emotional states, we employed the pre-scores for the dependent variable, age, and sex as covariates. In the GLM for measuring biomarkers using blood samples, we used the pre-scores for the dependent variable (age and sex) as covariates. In addition, to check an absorbed level of SFN, we conducted permutation GLM (SFN vs. placebo) for SFN-NAC urinary levels. We performed all GLMs with permutation tests using the ‘‘lmp’’ function in the lmPerm package.^2^ The permutation GLM was the same as in previous studies (Nouchi et al., 2020a,2021a,2021b; Kawata et al., 2021). Finally, based on our hypotheses, we harnessed a gatekeeping procedure based on Holm methods to adjust all of the pooled *p*-values. We carried out the gatekeeping procedure using the “pargateadjp” in multxpert package. We deemed the adjusted value *p* < 0.05 to be significant for multiple comparison methods.

## Results

We checked participants’ adherence to intake of supplements using a permutation test. The analysis did not reveal any significant differences between groups (*p* > 0.05) for the number of supplement-intake days (maximum = 84 days: SFN (*mean* = 83.9, *SD* = 0.4) or the placebo (*mean* = 83.9, *SD* = 0.2). In addition, we ensured there was no significant difference of the baseline measures between the groups (Table 1).

We analyzed the data based on the ITT (intention to treat) rule. For cognitive functioning, we found significant group differences in SS (*t* = 2.14, *uncorrected p* = 0.01, *adjusted p* = 0.03; Table 2). The processing speed performance of the SFN group improved compared to the placebo group (Table 2). For mood state, we found significant group differences in TMD (*t* = 2.15, *uncorrected p* = 0.01, *adjusted p* = 0.03; Table 2). Moreover, supplementary analyses for the subscale of POMS2 indicated that SFN reduced A-H (*t* = 1.53, *uncorrected p* = 0.02) and C-B (*t* = 1.51, *uncorrected p* = 0.02) compared to the placebo (see Supplementary Table 2).

**TABLE 2:** Baseline and change scores in cognitive functions and mood states in each group.

	Baseline score			Change score			
		
	PL	SFN	*p*-value	PL	SFN	*p*-value	Adjusted *p*-value
Cd	74.88	73.54	0.24	2.54	3.28	0.33	0.83
(Processing speed)	(14.26)	(14.09)		(6.64)	(7.37)		
SS	37.46	37.17	0.62	1.29	1.99	0.01	0.03
(Processing speed)	(7.34)	(5.79)		(4.76)	(3.83)		
ST	31.85	31.33	1.00	3.08	2.92	0.45	1
(Executive function)	(9.81)	(9.44)		(5.90)	(8.15)		
rST	44.79	45.68	0.37	2.50	2.90	0.26	1
(Executive function)	(9.13)	(7.75)		(4.65)	(5.63)		
TMT	46.08	43.32	0.40	–1.67	–0.93	0.36	1
(executive function)	(29.53)	(18.99)		(32.98)	(25.46)		
DS	17.21	17.04	0.52	0.56	0.07	0.08	0.2
(Verbal short-term memory)	(3.21)	(3.65)		(2.97)	(1.80)		
LNS	11.39	11.61	0.60	–0.08	–0.30	0.13	1
(Working memory capacity)	(2.23)	(2.70)		(2.32)	(2.25)		
LM immediately	22.99	24.31	0.27	2.51	2.41	0.50	1
(verbal episodic memory)	(6.74)	(7.06)		(4.34)	(4.73)		
LM delay	19.01	20.00	0.36	2.83	3.72	0.13	1
(Verbal episodic memory)	(7.36)	(7.60)		(4.71)	(5.69)		
DM	7.06	6.99	0.41	0.21	–0.03	0.12	1
(visual episodic memory)	(1.54)	(1.41)		(1.54)	(1.87)		
VPA	13.85	13.40	0.86	0.61	1.23	0.02	1
(Visual episodic memory)	(3.50)	(3.76)		(2.88)	(3.13)		
VR	33.67	34.08	0.94	0.83	0.32	0.08	1
(Visual episodic memory)	(4.58)	(3.30)		(3.72)	(3.17)		
TMD	–0.54	–1.44	0.76	1.60	–0.11	0.01	0.03
(Mood state)	(9.11)	(8.78)		(9.40)	(6.95)		
WHO-5	17.60	16.79	0.37	0.36	0.82	0.64	1
(Quality of life)	(3.85)	(3.14)		(3.33)	(3.04)		

Standard deviation (SD) in parentheses. SFN, sulforaphane; PL, placebo; Cd, digit symbol coding; SS, symbol search; ST, Stroop task; rST, reverse Stroop task; TMT, trail making test; DS, digit span; LNS, letter number sequence; LM, logical memory; DM, design memory; VPA, visual paired associates; VR, visual reproduction; TMD, total mood disturbance; WHO-5, World Health Organization-five wellbeing index.

**FIGURE 2:**
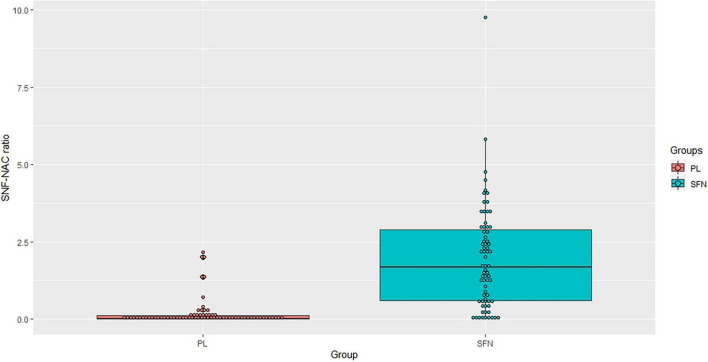
SNF-NAC ratio in each group.

For blood biomarkers, we did not find any significant group differences in oxidative stress (HO-1 and GST), inflammation (TNF-α), or neural plasticity (BDNF) (Table 3). However, we confirmed that the SFN group showed a greater SFN-NAC level compared to the placebo group (*t* = 8.68 *uncorrected p* < 0.01, *adjusted p* < 0.01, Figure 2).

**TABLE 3:** Baseline and change scores in biomarkers in each group.

	Baseline score			Change score			
		
	PL	SFN	*p*-value	PL	SFN	*p*-value	Adjusted *p*-value
GST	9.09	8.90	0.99	4.63	3.35	0.18	1
	(6.75)	(6.40)		(15.54)	(14.25)		
HO-1	0.64	0.45	0.35	–0.13	–0.04	0.09	1
	(1.53)	(0.32)		(0.82)	(0.24)		
TNF-α	0.44	0.41	0.42	–0.05	0.03	0.20	1
	(0.31)	(0.29)		(0.29)	(0.33)		
BDNF	26.55	26.28	0.78	–2.25	–2.80	0.99	1
	(6.77)	(7.28)		(5.80)	(7.95)		
SFN-NAC	–	–	–	0.18	1.94	<0.01	<0.01
	–	–	–	(0.46)	(1.67)		

Standard deviation (SD) in parentheses. SFN, sulforaphane; PL, placebo; GST, glutathione S-transferase; HO-1, heme oxygenase-1; TNF-α, tumor necrosis factor-α; BDNF, brain derived neurotrophic factor; SFN-NAC, sulforaphane N-acetyl-L-cysteine. SFN-NAC was only measured after the intervention period.

## Discussion

We aimed to reveal the beneficial effects of SFN intake on cognitive functioning and mood state in healthy older adults. We formulated three hypotheses related to cognitive functioning, mood state, and biomarkers. We expected that SFN intake would improve processing speed, decrease negative mood, and alter biomarkers. We derived three key findings corresponding to these hypotheses. For cognitive functioning, the SFN group exhibited a significant improvement in processing speed performance (measured by SS) compared to the placebo group. For emotional states, the SFN group significantly decreased negative mood (measured by TMD) compared to the placebo group. For the biomarkers, we found significant group differences in urinary SFN-NAC levels. In contrast, we did not observe any significant changes in the other biomarkers. We separately discuss each outcome below.

First, we noted a beneficial impact of SFN on processing speed performance. This result partially supports our hypothesis; it is consistent with previous cohort studies and RCTs. For example, one cohort study reported that people who consumed a large amount of cruciferous vegetables had better processing speed performance (Nurk et al., 2010). In addition, this result was consistent with a RCT using SFN intake and video game training. The previous study used 4 groups with 2 (nutrition factor; SFN intake or placebo intake) by 2 (video game factor: brain training game or puzzle game) factorial design. The previous study revealed both brain training and puzzle games groups with SFN intake led to a significant improvement of processing speed performance in healthy older adults (Nouchi et al., 2021a). In the previous study, it did not exclude an effect of playing video gaming. However, the current study is the first to demonstrate direct evidence that SFN intake alone enhances processing speed in healthy older adults. Taken together, these findings indicate that SFN intake can produce beneficial effects for processing speed performance.

Second, SFN intake decreased negative mood (measured by TMD). In addition, supplementary analyses for the subscale of POMS revealed that SFN intake selectively reduced the A-H and C-B scores. These results also support our hypothesis. The current finding is in line with previous evidence from animal and patient studies. Prior animal studies found that SFN treatment suppressed depression-like behaviors in mice (Wu et al., 2016; Zhang et al., 2017; Ferreira-Chamorro et al., 2018). Moreover, past studies on autism spectrum disorder (ASD) demonstrated that SFN reduced aberrant behaviors related to negative mood (Singh et al., 2014; Bent et al., 2018). The present study can expand existing findings to demonstrate that SFN intake can reduce the TMD score in healthy older adults.

For the biomarkers, we noted significant group differences in urinary SFN-NAC levels, but not in other biomarkers. This result partially supports our hypothesis related to SFN-NAC. The urinary SFN-NAC level is an indicator of SFN absorption. The increased level of SFN-NAC in the SFN group confirmed the compliance with taking the SFN or placebo supplements. In addition, we replicated the previous work, which showed increasements in SFN-NAC levels after SFN intake combined with cognitive training (Nouchi et al., 2021a). This study used the same method (participants, SFN supplements, intervention period) as the previous study. Taken together, this indicates that 12 weeks of intervention using SFN supplements containing 30 mg of glucoraphanin is enough to increase SFN absorption in healthy older adults.

We did not observe any significant effects of SFN on the blood biomarkers of antioxidant response, neural plasticity, or the neuroinflammation blood parameter. These results do not support our hypothesis. However, past human studies discovered that SFN intake did not change the antioxidant response level of HO-1 (Wise et al., 2016), the neuroinflammatory parameters of C-reactive protein (e.g., interleukin-6, interleukin-8) (Wise et al., 2016), or neural plasticity parameters (e.g., BDNF) (Shiina et al., 2015). In contrast, a prior study revealed a positive impact of SFN on antioxidant response, measured using GST (Egner et al., 2014). It should note that there are several methodological differences (biomarkers, intervention period, and participants) between the current study and the aforementioned ones.

It is important to consider the biological mechanism of SFN’s beneficial effects on cognitive functioning and mood state. SNF activates the Nrf2 antioxidant response, but may also have Nrf2-independent actions. The anti-oxidative and anti-inflammatory properties of SFN are key functions in explaining improvements in cognitive functioning and mood state. SFN has anti-oxidative and anti-inflammatory functions (Juge et al., 2007). Previous studies showed that the inflammation and oxidative stress levels were associated with cognitive functioning (Sartori et al., 2012; Hajjar et al., 2018) and negative mood state (Salim, 2014; Jones et al., 2020). It has been hypothesized that SFN intake will reduce oxidative and inflammatory levels, thereby enhancing cognitive functioning and mood state. However, our results do not directly support this hypothesis since we found no significant changes in the biomarkers of the antioxidant and anti-inflammatory levels following SFN intake. As mentioned before, there are some methodological limitations. It is difficult to conclude whether SFN’s anti-oxidative and anti-inflammatory properties can definitively explain improvements in cognitive functioning and mood. Hence, to confirm this hypothesis, future research should investigate the effects of SFN’s antioxidant and anti-inflammatory properties on cognitive functioning and mood using other populations and biomarkers.

Other proposed biological mechanisms include changes in neurotransmitter release and mitochondrial quality control (Huang et al., 2019). It is well known that neurotransmitters are associated with cognitive functioning and negative mood (Jenkins et al., 2016). In addition, the dysfunction of mitochondrial quality control is an essential hallmark of age-related cognitive decline (Apaijai et al., 2020) and negative mood, such as depressive symptoms (Allen et al., 2018). Previous studies, using 12-week periods of SFN intake for children with ASD, reported a significant correlation between improved clinical symptoms and a change in several urinary neurotransmitter-related metabolites, such as serotonin and homovanillate (HMV), which represent the normal end product of dopamine degradation (Bent et al., 2018). In addition, several previous animal studies found effects of SFN on brain mitochondria (Jardim et al., 2020). Thus, it is possible for changes in neurotransmitter release and modulations in mitochondrial quality control *via* SFN intake to enhance cognitive functioning and alleviate negative mood. However, we did not measure the functions of neurotransmitters or mitochondria. To prove these hypotheses, future research should investigate the role of neurotransmitters and mitochondria in boosting cognitive functioning and mood.

This study has some limitations. First, the intervention period: We set the length of the intervention period (12 weeks) based on a prior study (Nouchi et al., 2021a). However, it is important to examine whether shorter or longer SFN intervention periods would have positive outcomes for cognitive functioning and emotional states. In addition, a peak level of some biomarkers would occur earlier in the intervention period. The level of the biomarkers would be reduced over the intervention period. Therefore, it would be also important to check the biomarker multiple times. Second, the participants were healthy older adults. To generalize the findings, it is necessary to test whether SFN intake would have beneficial effects for young and middle-aged adults as well. Third, for cognitive functions, we found the significant improvements in VPA in the SFN group compared to the placebo group without adjustment of the *p*-value. However, the result did not survive after Bonferroni correction. It indicates that SFN would have a possibility to improve the visual episodic memory performance in older adults. Further study should be needed to evaluate the beneficial effect of SFN on the visual episodic memory. Finally, we did not control the amount of SFN intake from vegetables consumed daily before the intervention. The baseline difference in SFN intake can affect improvements in cognitive functioning and mood state. In future research, the amount of SFN intake should be controlled.

## Conclusion

We explored the beneficial effects of SFN intake on cognitive functioning and mood in healthy older adults. We found that 12 weeks of SFN intake boosted processing speed, reduced overall negative mood, and increased the SFN-NAC level compared to the placebo group, whose members took supplements without SFN. Although we did not find any significant changes in antioxidant response, neural plasticity, or the neuroinflammation blood parameter, these results indicate that nutrition interventions using SFN can have positive effects on cognitive functioning and mood in healthy older adults.

## Data availability statement

The raw data supporting the conclusions of this article will be made available by the authors, without undue reservation.

## Ethics statement

The studies involving human participants were reviewed and approved by the Ethics Committee of KAGOME CO., LTD. (Ref. 2020-R11) and the Ethics Committee of Nihonbashi Cardiology Clinic (Ref. NJI-020-10-01). The patients/participants provided their written informed consent to participate in this study.

## Author contributions

RN and QH: conceptualization, methodology, investigation, data curation, writing—original draft preparation, visualization, and project administration. RN: formal analysis and funding acquisition. RN, QH, YU, HS, and RK: writing—review and editing. RK: supervision. All authors have read and agreed to the published version of the manuscript.

## Footnotes

1
http://www.consort-statement.org/home/

2
http://cran.r-project.org/web/packages/lmPerm/index.html
