# Supplementary material for: Effects of sulforaphane intake on processing speed and negative moods in healthy older adults: Evidence from a randomized controlled trial
Source: Front Aging Neurosci. 2022 Jul 29;14:929628. doi: 10.3389/fnagi.2022.929628 (PMC9372582; doi:10.3389/fnagi.2022.929628)
Supplement: Supplementary file 1 [file Data_Sheet_1.DOCX]

**File S1: Description of details of cognitive functional measurements**

To assess participants’ processing speed, we used digit symbol coding (Cd) and symbol search (SS) from the Wechsler Adult Intelligence Scale, 3^rd^ edition (WAIS-III) [1]. We reproduced the following descriptions of Cd and SS from our earlier report [2]: “For Cd, the participants were shown a series of symbols that were paired with numbers. Using a key within a 120-s time limit, participants drew each symbol under its corresponding number. The primary measure of this test was the number of correct answers. In SS, participants visually scanned two groups of symbols (a target group and a search group) and indicated whether either of the target symbols matched any symbol in the search group. Participants responded to as many items as possible within a 120-s time limit. The primary measure of this test was the number of correct answers.”

To establish executive functioning, we used the Stroop task (ST) and the reverse Stroop task (rST) [3], as well as the trail making test (TMT) [4]. We reproduced the following descriptions of ST and TMT from our earlier report [2]: “In the ST, in the leftmost of six columns, a word naming a color was printed in another color (e.g., ‘red’ was printed in blue letters); the other five columns contained words naming colors. Participants were required to check the column containing the word naming the color of the word in the leftmost column. In the rST, in the leftmost of six columns, a word naming a color was printed in another color (e.g., ‘red’ was printed in blue letters); the other five columns were filled with five different colors, from which participants were required to check the column with the color matching the word written in the leftmost column. In each task, participants were instructed to complete as many of these exercises as possible in 1 min. The primary measure for this task was the number of correct items” [2]. Further, “TMT has been employed widely as a measure of executive function. TMT consists of two parts (TMT-A and TMT-B). TMT-A requires participants to link in ascending order series of 25 numbers (1–2–3 …) randomly distributed in space. TMT-B is similar, although instead of just linking numbers the subject must alternately switch between a set of numbers (1–13) and a set of letters (A–L), again linking in ascending order (1–A–2–B …). The primary measure of this test is the amount of time (seconds) required to complete the task.”[5]. We used the Δ in the TMT score (part B minus part A).

To measure short-term memory and working memory performance, we used the digit span (DS) and letter number sequence (LNS), which are subtests of the WAIS-III [1]. We reproduced the following descriptions of DS and LNS from our earlier report [2][6]: “For the DS-forward, participants repeated numbers in the same order as they were read aloud by the examiner. For the DS-backward, participants repeated numbers in the reverse order of that read aloud by the examiner. In both the tasks, the examiner read a series of number sequences which the participant was required to repeat in either the forward or reverse order” [2]. The primary measure of this test is the correct answer. The maximum score in the DS is 30. For LNS, “the examiner read a combination of letters and numbers; then participants were asked to recall numbers first in ascending order, followed by the letters in Japanese alphabetical order. If participants responded with letters first, followed by numbers, but with all of them in the correct sequence, credit was awarded. LNS begins with the simplest level of a three-letter number sequence. There are five sets of letters and numbers in increasing length, and each set consists of three trials (total 15 trials). The maximum raw score is 15.”

To measure verbal episodic memory performance, we used the logical memory (LM) subtest of the Wechsler Memory Scale-Revised (WMS-R) [7]. To examine visual episodic memory, we used design memory (DM), visual paired associates (VPA), and visual reproduction (VR). We reproduced the description of LM from a prior study[2]. “LM consists of two short-paragraph-length stories (Story A and Story B). For the LM, participants were required to memorize one of the two stories. The stories were scored in terms of the number of story units recalled, as specified in the WMS-R scoring protocol. We used either Story A or Story B. The primary measure for this task was the number of correct story units recalled” [2]. In the present study, we employed immediately and delayed recall memory performance. For DM, which has four tests, participants are asked to memorize abstract pattern figures. For test 1, they memorize one abstract pattern figure for 5 seconds. Then, they must select the same abstract pattern figure from three abstract pattern figures. For tests 2, 3, and 4, they memorize three abstract pattern figures for 15 seconds. Next, participants must select the three same abstract pattern figures from nine abstract pattern figures. The primary measure of DM is the total number of correct answers (maximum score=10). For VPA, which entails the use of six pairs of figures and colored patches, participants are asked to memorize a pair of figures and colored patches. First, each pair of figures and colors is presented for 3 seconds. Afterward, only figures are presented; during this time, participants are asked to select the colored patch that matches the figures. The primary measure of VPA is the total number of correct answers (maximum score=18). For VR, which has four tests, the participants are asked to memorize geometric shapes and to draw them. Each geometric shape is presented for 10 seconds. Next, the participants are asked to draw the geometric shape, which is evaluated based on scoring criteria. The maximum score is 41.

1. Wechsler, D.A. *Wechsler Adult Intelligence Scale Third Edition*; The Psychological Corporation: San Antonio, TX., 1997;

2. Nouchi, R.; Taki, Y.; Takeuchi, H.; Hashizume, H.; Nozawa, T.; Sekiguchi, A.; Nouchi, H.; Kawashima, R. Beneficial Effects of Reading Aloud and Solving Simple Arithmetic Calculations (Learning Therapy) on a Wide Range of Cognitive Functions in the Healthy Elderly: Study Protocol for a Randomized Controlled Trial. *Trials* **2012**, *13*, 32, doi:10.1186/1745-6215-13-32.

3. Hakoda, Y.; Watanabe, M. *Manual for New Stroop Test II*; Toyo Physical.: Fukuoka, Japan, 2004;

4. Japan Society for Higher Brain Dysfunction, B. function test committee *Trail Making Test, Japanese Edition (TMT-J)*; Shinkoh Igaku Shuppansha Co., Ltd., 2019;

5. Nouchi, R.; Taki, Y.; Takeuchi, H.; Hashizume, H.; Akitsuki, Y.; Shigemune, Y.; Sekiguchi, A.; Kotozaki, Y.; Tsukiura, T.; Yomogida, Y.; et al. Brain Training Game Improves Executive Functions and Processing Speed in the Elderly: A Randomized Controlled Trial. *PLoS One* **2012**, *7*, e29676, doi:10.1371/journal.pone.0029676.

6. Nouchi, R.; Taki, Y.; Takeuchi, H.; Hashizume, H.; Nozawa, T.; Kambara, T.; Sekiguchi, A.; Miyauchi, C.M.; Kotozaki, Y.; Nouchi, H.; et al. Brain Training Game Boosts Executive Functions, Working Memory and Processing Speed in the Young Adults: A Randomized Controlled Trial. *PLoS One* **2013**, *8*, e55518, doi:10.1371/journal.pone.0055518.

7. Wechsler, D.A. *Wechsler Memory Scale Revised*; The Psychological Corporation: San Antonio, TX., 1987;
