# Supplementary material for: Effects of sulforaphane intake on processing speed and negative moods in healthy older adults: Evidence from a randomized controlled trial
Source: Front Aging Neurosci. 2022 Jul 29;14:929628. doi: 10.3389/fnagi.2022.929628 (PMC9372582; doi:10.3389/fnagi.2022.929628)
Supplement: Supplementary file 2 [file Data_Sheet_2.DOCX]

**File S2: Description of details of urine biomarkers**

To determine whether SFN was absorbed into the body, the excreted level of SFN-NAC, a major metabolite of SFN, was analyzed in urine samples collected from the participants 10–12 hours after their final intake of the SFN or placebo supplement. The time was when urinary excretion of isothiocyanate metabolites would have been expected to reache a maximum level　[1]. We reproduced the description of the urine analysis from a previous study [2]: “Urine samples (stored at -30°C) were thawed on ice and centrifuged (14000 × g, 15 min, 4°C) to precipitate proteins. The supernatants were filtered through Ultrafree®-MC, GV 0.22-μm centrifugal filters (Merck Millipore, MA), and the filtrates (25 μL) along with the internal standard iberin were subjected to HPLC-MS/MS analysis (a Shimadzu 20A HPLC system [Shimadzu, Japan] coupled to an LCQ Fleet electrospray ionization (ESI) ion trap mass spectrometer [Thermo Scientific]) in duplicate). The quantitation of SFN-NAC was based on a 5-point standard curve, and the internal standard was dependent on the presence of two peaks monitored with the expected area ratio. Finally, the urinary levels of SFN-NAC were standardized to creatinine levels, which were determined using a commercial kit (Exocell, PA) according to the manufacturer’s instructions.”

1. Ushida, Y.; Suganuma, H.; Yanaka, A. Low-Dose of the Sulforaphane Precursor Glucoraphanin as a Dietary Supplement Induces Chemoprotective Enzymes in Humans. *Food and Nutrition Sciences* **2015**, *06*, 1603–1612, doi:10.4236/fns.2015.617165.

2. Nouchi, R.; Hu, Q.; Saito, T.; Kawata, N.Y. dos S.; Nouchi, H.; Kawashima, R. Brain Training and Sulforaphane Intake Interventions Separately Improve Cognitive Performance in Healthy Older Adults, Whereas a Combination of These Interventions Does Not Have More Beneficial Effects: Evidence from a Randomized Controlled Trial. *Nutrients* **2021**, *13*, 352, doi:10.3390/nu13020352.
