# Supplementary material for: Effects of sulforaphane intake on processing speed and negative moods in healthy older adults: Evidence from a randomized controlled trial
Source: Front Aging Neurosci. 2022 Jul 29;14:929628. doi: 10.3389/fnagi.2022.929628 (PMC9372582; doi:10.3389/fnagi.2022.929628)
Supplement: Supplementary file 4 [file Table_2.DOCX]

**Supplementary Table 2. Baseline and change scores in subscales of POMS in each group**

| Baseline score | | | | Change score | | |
| --- | --- | --- | --- | --- | --- | --- |
|  | P | SFN | p value | P | SFN | p value |
| A-H | 1.82 | 2.06 | 0.76 | 0.28 | -0.13 | 0.02 |
|  | (2.13) | (2.20) |  | (1.79) | (1.90) |  |
| C-B | 1.68 | 1.31 | 0.64 | 0.39 | -0.08 | 0.02 |
|  | (1.93) | (1.59) |  | (2.58) | (1.42) |  |
| D | 1.11 | 0.83 | 1.00 | 0.26 | 0.23 | 0.43 |
|  | (1.38) | (1.37) |  | (1.81) | (1.34) |  |
| F-I | 2.40 | 1.81 | 0.16 | 0.19 | 0.13 | 0.49 |
|  | (2.57) | (1.97) |  | (2.35) | (1.77) |  |
| T-A | 2.96 | 2.51 | 0.16 | 0.42 | 0.31 | 0.78 |
|  | (2.41) | (2.10) |  | (2.64) | (2.08) |  |
| V | 10.51 | 9.96 | 0.45 | -0.06 | 0.56 | 0.16 |
|  | (4.33) | (4.21) |  | (3.30) | (3.48) |  |
| F | 10.83 | 10.47 | 0.90 | 0.26 | 0.49 | 0.27 |
|  | (3.82) | (3.81) |  | (2.64) | (2.63) |  |

Note: Standard deviation (SD) in parentheses. SFN: sulforaphane, P: placebo, A-H: anger–hostility, C-B: confusion–bewilderment, D: depression–dejection, F-I: fatigue–inertia, T-A: tension–anxiety, V: vigor–activity, F: friendliness.
